# Supplementary material for: DMRforPairs: identifying Differentially Methylated Regions between unique samples using array based methylation profiles
Source: BMC Bioinformatics. 2014 May 15;15:141. doi: 10.1186/1471-2105-15-141 (PMC4046028; doi:10.1186/1471-2105-15-141)
Supplement: Additional file 2 — DMRforPairs output for the comparison of A431-MCF7 and NA17018-NA17105. Please start from the HTML files in each folder. Available via the BMC Bioinformatics website. [file 1471-2105-15-141-S2.zip › 1394847754114233_MOESM2_ESM/NA17105M_NA17018F/figures/20992.pdf]

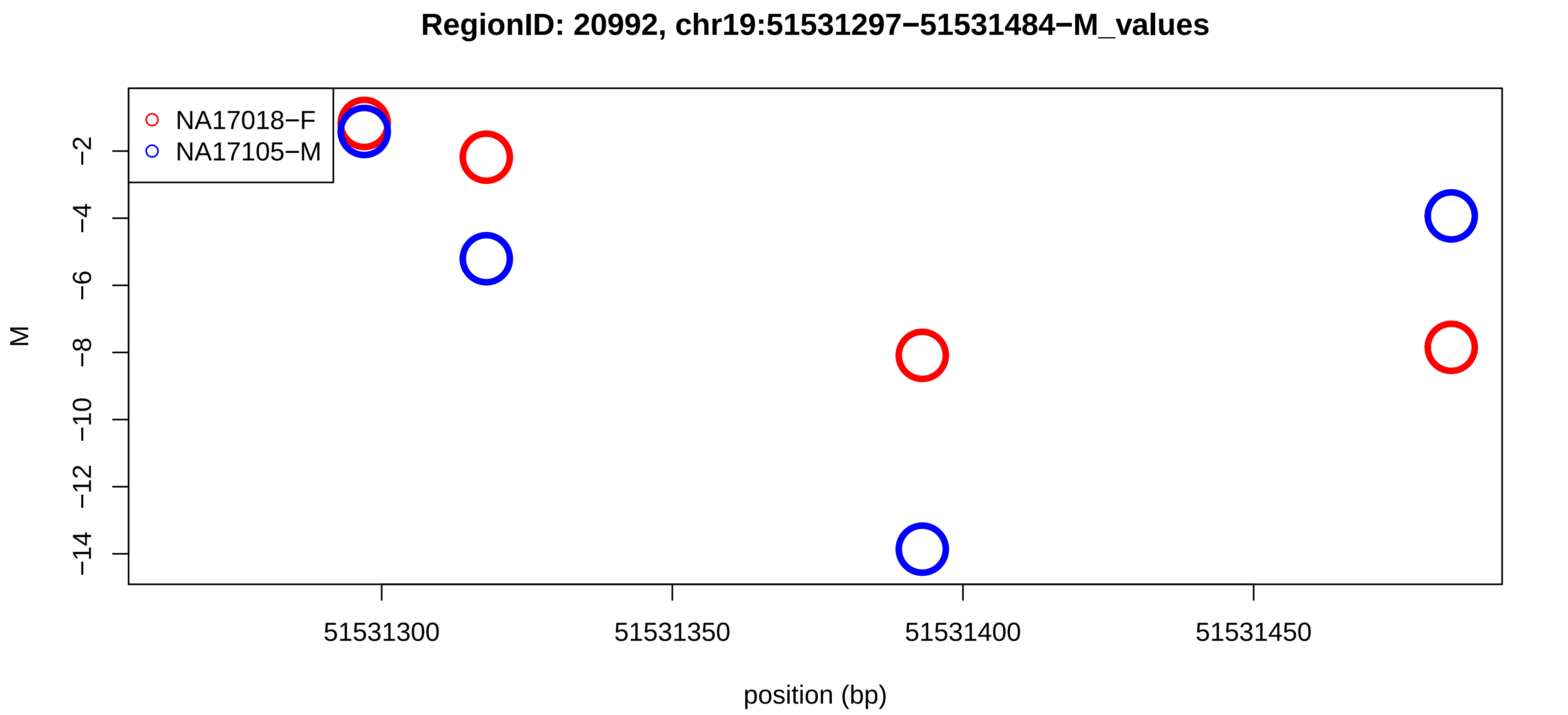
RegionID: 20992, chr19:51531297−51531484−M_values
NA17018−F
NA17105−M
M
−2
−4
−6
−8
−10
−12
−14
51531300
51531350
51531400
51531450
position (bp)

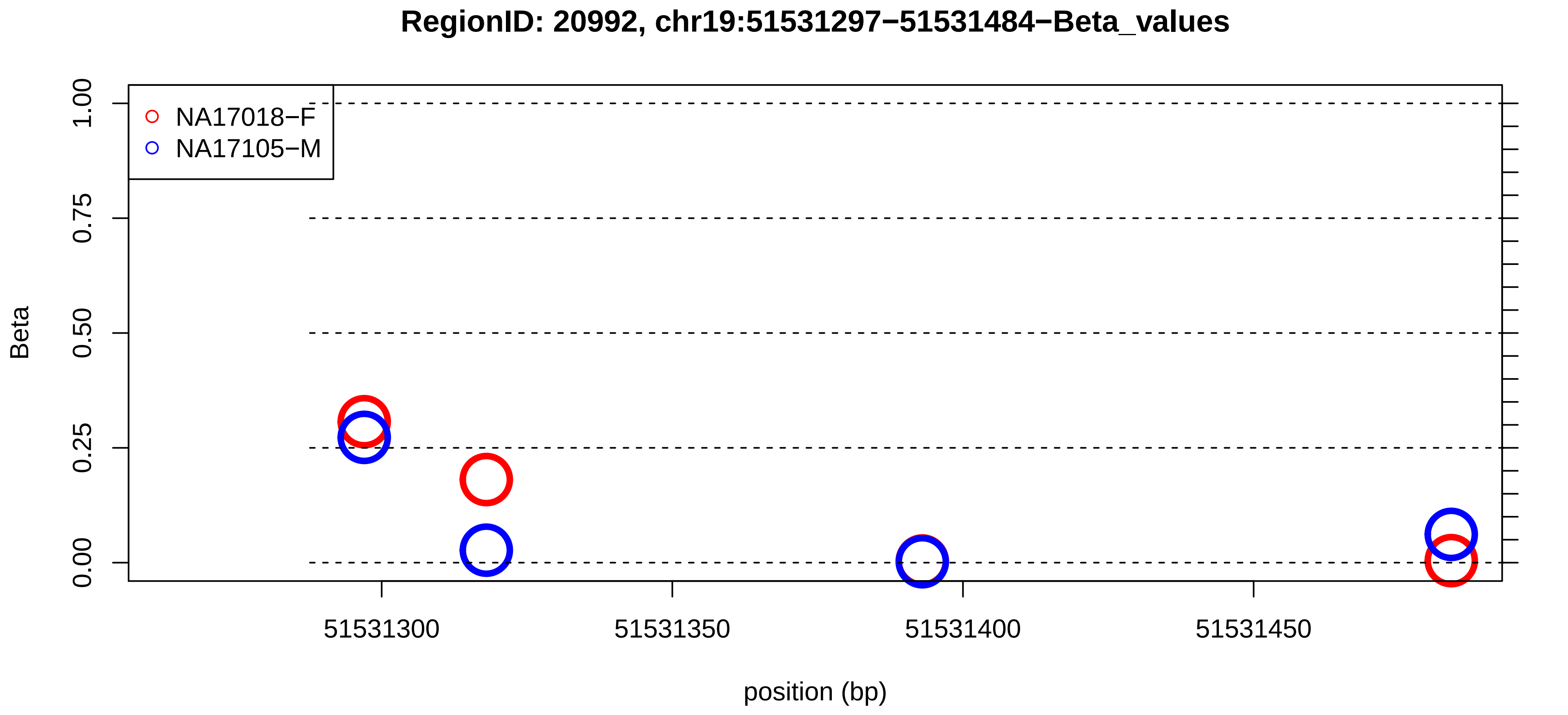
RegionID: 20992, chr19:51531297−51531484−Beta_values
NA17018−F
NA17105−M
Beta
0.00
0.25
0.50
0.75
1.00
51531300
51531350
51531400
51531450
position (bp)
